# New Drugs, Appliances, and Things Medical

**Published:** 1892-02-27

**Authors:** 


### NEW DRUGS, APPLIANCES, AND THINGS MEDICAL.

[All preparations' appliances, novelties, etc., of which a notice is desired, should be sent for The Editor, to care of The Manager, 140, Strand, London, W.C.]

### PEPSALIA.

There is little doubt that indigestion is the increasing complaint of the age. Any means to abate the evil should earn the gratitude of the public. Messrs. Stern, of Gray's Inn Road, have provided in "pepsalia" a most excellent preparation for the assistance of digestion. Pepsalia is a digestive in the form of a table salt, of which it is an agreeable substitute, and thus the irksomeness of medicine-taking is avoided, whilst the beneficial effect on those suffering from ordinary forms of dyspepsia is indisputable. Pepsalia is becoming increasingly and deservedly popular, whilst the verdict of the analyst and the profession as to the harmless nature of its components is most re-assuring. The preparation acts on the food alone, and is therefore not injurious to the stomach. It is sold in bottles at 1s., 2s., and 3s. each.

### PREPARATIONS OF PURE BEEF.

The Pure Beef Company, Great Tower Street, have provided us with samples of their preparations, to all of which we have given a careful trial. The company have placed their specialities within the reach of those unable to afford the more expensive forms of meat essences, whilst they claim for them all the qualities possessed by their rivals. Their essence of beef and concentrated beef tea are most carefully prepared for the use of the sick room. For household purposes they offer the public clear and thick stocks as foundations for any variety of soups desired, also extracts of beef, and what is moreover a novelty, their concentrated beef à la mode. The beef à la mode is a savoury dish, which will prove valuable, especially to country householders as a safeguard against the irregular visits of the butcher. Lastly, the meat lozenges are invaluable to tourists and public speakers. All the extracts contain a high percentage of nutritious fibrin.

### SALVINE.

We have already spoken in praise of salvine as a dentifrice, and frequently find our opinion endorsed by those who have made a trial of it. We are glad to speak with equal approbation of the other toilet articles prepared at the Salvine Depot, 3, Oxford Street. The scientific soap, which is super fatted de-alkalised and de-hydrated, is of excellent quality, and most agreeable to the skin. The shaving soap can be equally recommended. Salvine toilet powder, a very fine preparation, is sold in three tints.

